# Female Mice Have Higher Angiogenesis in Perigonadal Adipose Tissue Than Males in Response to High-Fat Diet

**DOI:** 10.3389/fphys.2018.01452

**Published:** 2018-10-23

**Authors:** Martina Rudnicki, Ghoncheh Abdifarkosh, Omid Rezvan, Emmanuel Nwadozi, Emilie Roudier, Tara L. Haas

**Affiliations:** Angiogenesis Research Group, School of Kinesiology and Health Science and the Muscle Health Research Centre, York University, Toronto, ON, Canada

**Keywords:** adipose tissue, diet-induced obesity, microvascular, sex dimorphism, skeletal muscle

## Abstract

**Background:** Impaired capillary growth (angiogenesis) in skeletal muscle and adipose tissue contributes to the development of metabolic disorders in obese males. This association remains unexplored in females, despite mounting evidence that endothelial cells have sex-specific transcriptional profiles. Therefore, herein we assessed whether males and females show distinct angiogenic capacities in response to diet-induced obesity.

**Methods:** Age-matched male and female mice were fed normal chow or high-fat obesogenic diets for 16 weeks. At the end of diet period, systemic glucose disposal was assessed as well as insulin sensitivity of skeletal muscle and visceral adipose tissue. Capillary content and the expression of angiogenic regulators were also evaluated in these tissues.

**Results:** When placed on a high-fat diet, female mice gained less weight than males and showed a metabolic phenotype similar to NC-fed mice, contrasting with the impaired whole-body glucose metabolism observed in high-fat-fed males. However, high-fat-feeding elevated serum lipid levels similarly in male and female mice. Although skeletal muscle of high-fat–fed female mice had higher insulin sensitivity than male counterparts, no sex difference was detected in muscle capillarization. Metabolic functions of perigonadal white adipose tissue (pgWAT) were retained in high-fat-fed females, as evidenced by smaller adipocytes with preserved insulin sensitivity, greater responsiveness to isoproterenol, higher expression of *Adiponectin* and a lower ratio of *Leptin:Adiponectin* mRNA. An enhanced browning phenotype was detected in HF-fed female adipocytes with upregulation of *Ucp1* expression. PgWAT from high-fat-fed females also showed augmented capillary number and expression of endothelial cell markers, which was associated with elevated mRNA levels of pro-angiogenic mediators, including vascular endothelial growth factor A (*Vegfa*) and its receptor (*Vegfr2*), the Notch ligand Jagged-1 (*Jag1*) and Angiopoietin-2 (*Angpt2)*.

**Conclusion:** Taken together, our findings provide novel evidence that visceral adipose tissue of female mice display greater levels of pro-angiogenic factors and vascularity than males in response to high-fat diet. This phenotype is associated with preserved metabolic homeostasis at both tissue and systemic levels. Our study discloses that a thus-far-unappreciated sex-specific difference in the regulation of adipose angiogenesis may contribute to an individual’s susceptibility to developing adipose dysfunction and obesity-related metabolic disturbances.

## Introduction

Mounting evidence of clinically important sex-related differences in the development of obesity-driven disturbances is garnering research attention, particularly in light of the growing global public health burden of obesity. Increase in body mass index (BMI) is strongly associated with the incidence of obesity complications in both sexes (Whitlock et al., 2009; Khan et al., 2018). However, despite higher rates of obesity in women (Ng et al., 2014), men are diagnosed with obesity-related complications at lower BMI than women (Logue et al., 2011; Paul et al., 2012; Wannamethee et al., 2012; Steinarsson et al., 2018). Moreover, animal studies report that females are more resistant to develop obesity-related metabolic disorders than males (Hevener et al., 2002; Macotela et al., 2009; Grove et al., 2010; Medrikova et al., 2012). Although this sex-dimorphic pathophysiology has been linked to the greater expansion of visceral adipose tissue that occurs in males (Macotela et al., 2009; Tchernof and Despres, 2013), the mechanisms underlying sex-related differences in the development of diet-induced disturbances are ill-defined.

Capillary networks modulate tissue functions not only by regulating oxygen and nutrient supply (Pittman, 2013) but also by releasing angiocrine factors (Rafii et al., 2016) and controlling delivery of hormones and growth factors (Kolka and Bergman, 2012). To adequately support tissue growth and metabolic requirements, capillary networks expand through angiogenesis, where new blood vessels develop from those pre-existing within the tissue (Potente et al., 2011). As a result, it has long been recognized that appropriate density and distribution of capillary networks is vital for tissue adaptations to physiological and pathological challenges (Rivard et al., 1999; Pasarica et al., 2009; Sun et al., 2012).

Nutrient oversupply associated with a high-fat diet trigger metabolic adaptation within adipose tissue and skeletal muscle to increase tissue oxygen demand, which should stimulate vascular remodeling. In contrast, impaired angiogenesis is reported in both tissues with a sustained high-fat diet (Rivard et al., 1999; Pasarica et al., 2009). Importantly, reduced capillary number in skeletal muscle is correlated with decreased peripheral glucose utilization (Bonner et al., 2013) as well as whole-body insulin resistance (Lillioja et al., 1987; Prior et al., 2015; Nwadozi et al., 2016). Moreover, reduced adipose tissue vascularization is proposed as one of the main contributors to the pathogenesis of dysfunctional adipose tissue (Crewe et al., 2017), which leads to the development of obesity-induced metabolic complications. Conversely, increasing the capillary density in either skeletal muscle (Prior et al., 2015) or adipose tissue (Sun et al., 2012) improves not only peripheral but also whole-body metabolism. These findings imply that deficiencies in angiogenesis may underlie the disruption of homeostasis induced by obesogenic signals in the adipose tissue and skeletal muscle, contributing to the development of obesity-driven disorders.

Despite this prevalent idea, the association between impaired angiogenesis and obesity-related disturbances has been explored only in males. Notably, it was recently reported that the endothelial cells that line capillaries exhibit sexual dimorphism (Addis et al., 2014; Lorenz et al., 2015; Cattaneo et al., 2017). Endothelial cells from female donors display higher ability to form capillary-like tubes *in vitro* (Lorenz et al., 2015) as well as increased proliferative and migratory capacities (Addis et al., 2014). It was also shown that estradiol, the main female sex hormone, modulates angiogenesis via effects on endothelial cells (reviewed in Losordo and Isner, 2001), and regulates gene expression of vascular endothelial growth factor-A (VEGFA) – the master regulator of angiogenesis – in adipocytes and adipose tissue (Fatima et al., 2017). Furthermore, transcriptomic analysis of human skeletal muscle identified elevated levels of angiogenic factors in females, including VEGF-A (Lindholm et al., 2014), indicating that tissues from females could present higher angiogenic potential. However, sex-differences in the angiogenic response of endothelial cells to a high-fat diet have so far not been directly addressed.

Therefore, in this study, we investigated the hypothesis that male and female mice undergo different angiogenic responses to the obesogenic signals elicited by high-fat feeding, which could, in turn, contribute to the known sex-related differences in the development and severity of diet-induced metabolic disorders. Thus, we examined the vascular density of skeletal muscle and visceral adipose tissue as well as tissue functions and whole-body metabolism of male and female mice when subjected to the metabolic stress of a 16-week high-fat diet.

## Materials and Methods

### Animals and Study Design

At 5–8 weeks of age, age-matched female and male mice (FVB;B6) were randomly allocated to receive either a low-fat diet (*n* = 7 males and 8 females, 11% kcal from fat; #D12329, Research Diets) or a 58% high-fat diet (*n* = 7 males and 7 females, Surwit Diet, #D12331, Research Diets) for 16 weeks. Mice were maintained at a controlled temperature (22°C) and a 12-h light–dark cycle (light on from 0700 to 1900 h). Water and diet were provided *ad libitum*. Body weights and food intake were recorded weekly. After 16 weeks, mice were fasted for 2 h, euthanized under isoflurane anesthesia and tissues were removed, weighed and utilized as described below. Tests were conducted in all mice used for this study. However, samples or data points were excluded in the case of a technical equipment or human error that caused a sample to be poorly controlled. A second cohort of weight-matched adult male and female mice (FVB; B6, *n* = 4 males and 4 females) underwent HF feeding for 1 week and tissue collection for adipose vascularity and gene expression analyses. The study was conducted in accordance with the American Physiological Society’s guiding principles in the Care and Use of Animals, following protocols approved by the York University Committee on Animal Care.

### Systemic Metabolic Tests

Insulin tolerance tests (ITT) were conducted at week 14. Food was removed in the morning and ITT were performed in the afternoon. After 4 h fasting, insulin (i.p. 0.75U/kg BW Humalog; Lilly) was injected to mice. For the glucose tolerance test (GTT), performed at week 15, overnight fasting was conducted prior to administration of glucose (i.p. 1.75 g/kg BW). Blood glucose was measured by a glucometer (Freestyle Lite, Abbott Diabetes Care, ON, Canada) at 0, 20, and 40 min after insulin injection (for ITT) or at 0, 30, 60, 90, 120, and 180 min after glucose injection (for GTT).

### *In vivo* Skeletal Muscle Insulin Sensitivity

Insulin sensitivity of skeletal muscle was assessed *in vivo* after 16 weeks of diet using the extensor digitorum longus (EDL) muscle as previously described (Nwadozi et al., 2016). Briefly, 2-h-fasted mice were maintained under isoflurane anesthesia and one EDL muscle was removed and snap frozen, before receiving insulin (i.p. 0.12U, Humalog; Lilly). After 15 min, the contralateral EDL was removed and snap frozen and the phosphorylation state of Akt was assessed by Western blotting.

### *Ex vivo* Adipose Incubation

Perigonadal fat pads (pgWAT) isolated from 2-h-fasted mice (removed prior to i.p. insulin injection described above) were cut into ∼80 mg fragments, and pre-incubated with DMEM low glucose containing 1% fatty acid-free BSA for 30 min (37°C) before incubation in the absence or presence of insulin (25 mU/mL, Humalog; Lilly) or isoproterenol (10 μmol/L, #1747, Tocris Bioscience) for another 30 min. Adipose tissue explants were then snap-frozen in liquid nitrogen and the phosphorylation states of Akt or HSL were assessed by Western blotting.

### Western Blot

Total protein extraction from EDL and pgWAT was performed as previously described (Milkiewicz et al., 2011). The following polyclonal primary antibodies were used: Ser473-pAkt, Akt, Ser563-pHSL, HSL and α/β-tubulin (Cell Signaling Technology, #4058, #9272, #4139, #4107 and #2148, respectively) and β-actin (sc-47778, Santa Cruz Biotechnology). Secondary antibodies were goat anti-rabbit or anti-mouse IgG-horseradish peroxidase (Jackson ImmunoResearch Laboratories, # 111-035-003, 115-035-003, respectively). Membranes were developed using enhanced chemiluminescence (SuperSignal^TM^ Westpico, #34080, ThermoFisher Scientific) and densitometry analysis was performed with ImageJ Analysis Software (NIH).

### Muscle Histology

Transverse cryosections (10 μm thick) from EDL muscle were fixed in 3.7% formaldehyde and stained with fluorescein isothiocyanate-conjugated *Griffonia simplicifolia* Lectin-1 (1:100; #FL11101, VectorLabs) and Rhodamine Wheat Germ Agglutinin (1:1000; #RL1022, VectorLabs) to detect capillaries and to outline muscle fibers, respectively. Capillary-to-fiber (C:F) ratios were calculated as previously described (Milkiewicz et al., 2011).

### Imaging of Adipose Tissue

For whole-mount analysis of adipose tissue vascularization, small pieces of pgWAT were fixed in 4% formaldehyde and washed with PBS. pgWAT pieces were stained with BODIPY (0.25 μg/mL, #D3922, ThermoFisher Scientific) and rhodamine labeled *Griffonia simplicifolia* lectin (1:100, #RL1102, VectorLabs) to visualize adipocytes and microvessels, respectively. Images were captured at the focal plane containing the highest lectin signal, using a Zeiss LSM700 confocal microscope (N-Achromoplan 10x/0.25 objective; pinhole 71 μm) and identical gain settings for all samples. Microvascular content and branchpoint numbers were quantified from 3-4 10x fields of view per animal using Image J Analysis Software (NIH). For quantification of adipocyte size and capillary number, pieces of formaldehyde-fixed pgWAT were paraffin-embedded and sectioned (Toronto Centre for Phenogenomics). Hematoxylin and eosin-stained sections were imaged using a 4x objective. Image J was used to calculate adipocyte cross-sectional areas, based on 2-3 fields of view and a minimum of 500 adipocytes per mouse. For quantification of capillaries, sections were stained with fluorescein isothiocyanate-conjugated *Griffonia simplicifolia* Lectin-1 (1:100) and Rhodamine Wheat Germ Agglutinin (1:200). Images were acquired using a 10x objective on a Zeiss inverted microscope equipped with a digital cooled CCD camera, capturing 3–5 independent fields of view per mouse. Image J software was used to quantify the numbers of capillaries and adipocytes in corresponding *Griffonia* and Wheat Germ Agluttinin-stained images, respectively.

### Lipid Measurements

Fasting serum triglycerides and glycerol levels were determined using Triglyceride Colorimetric Assay kit (#10010303, Cayman Chemical Company) and Glycerol Assay Kit (#MAK117, Sigma-Aldrich), respectively.

### Isolation of Stromal-Vascular Fraction

A portion of pgWAT was digested with 0.5% Type I collagenase (#17100-017, ThermoFisher Scientific) for 30 min at 37°C with shaking. Centrifugation (300 × *g* for 5 min) was used to separate adipocytes from the stromal vascular fraction (SVF). Floating adipocytes and lipid layer were removed. The cell pellet was washed twice with Hepes Buffered Saline supplemented with 1% bovine serum albumin, dissolved in QIAzol Lysis Reagent (Qiagen) and stored to -80°C. Due to small amount of starting material for RNA isolation and poor-quality total RNA obtained after RNA isolation for some samples, this analysis was performed in a reduced sample size (*n* = 5 NC-males, 7 NC-females, 6 HF-males and 4 HF-females).

### RNA Extraction and Real-Time Quantitative PCR (qPCR)

Total RNA was isolated from EDL, pgWAT and SVF using RNeasy Mini Kits (Qiagen Inc.). Synthesis of cDNA was performed using the M-MLV reverse transcriptase (#M0253, New England Biolabs). mRNA levels were analyzed by real-time PCR on the Rotor-Gene Q platform (Qiagen Inc.) using Fast TaqMan Master Mix (#4444963 ThermoFisher Scientific) and TaqMan^®^ Assay-on-Demand primer sets for *Actb* (Mm04394036_g1), *Adipoq* (Mm00456425_m1), *Angpt1* (Mm00456503_m1), *Angpt2* (Mm00545822_m1), *Apln* (Mm00627688_g1), *Cidea* (Mm004 32553_m1), *Dll4* (Mm00444619_m1), *Esr1* (Mm00433149_m1), *Esr2* (Mm00599821_m1), *Jag1* (Mm00496902_m1), *Kdr* (Mm012 22421_m1), *Hprt1* (Mm00446968_m1), *Leptin* (Mm0043475 9_m1), *Nos3* (Mm00435217_m1), *Pecam1* (Mm00476712_m1) *Ppargc1a* (Mm00447183_m1), *Ucp1* (Mm01244861_m1), *Vegfa* (Mm00437306_m1), *Vwf* (Mm00550376_m1). Expression of each target gene was calculated relative to *Hprt1* or *Actb* levels and expressed as 2^-ΔCt^.

### Statistical Analysis

All results presented are mean ± standard error of the mean (SEM). Weekly caloric intake was determined between weeks 7 and 13. Metabolic efficiency was calculated as the energy intake consumed during weeks 7 and 13 divided by the body weight gain over the same period of time. Statistical analyses were performed using Prism 5 (GraphPad Software Inc.). Significance was established at *P* < 0.05, by two-way ANOVA, with factors of sex and feeding conditions. The effects of insulin injection on muscle insulin sensitivity and incubation of adipose tissue with insulin or isoproterenol were examined using repeated-measures ANOVA. *Post hoc* Bonferroni-corrected *t*-tests were performed when a statistically significant difference was found in the two-way ANOVA model. *P*-values are provided in each figure legend and indicated in figures as ^∗^*P* < 0.05, ^∗∗^*P* < 0.01, ^∗∗∗^*P* < 0.001.

## Results

### Female Mice Are More Resistant to the Development of Diet-Induced Obesity and Related Metabolic Abnormalities Than Male Mice

**FIGURE 1:**
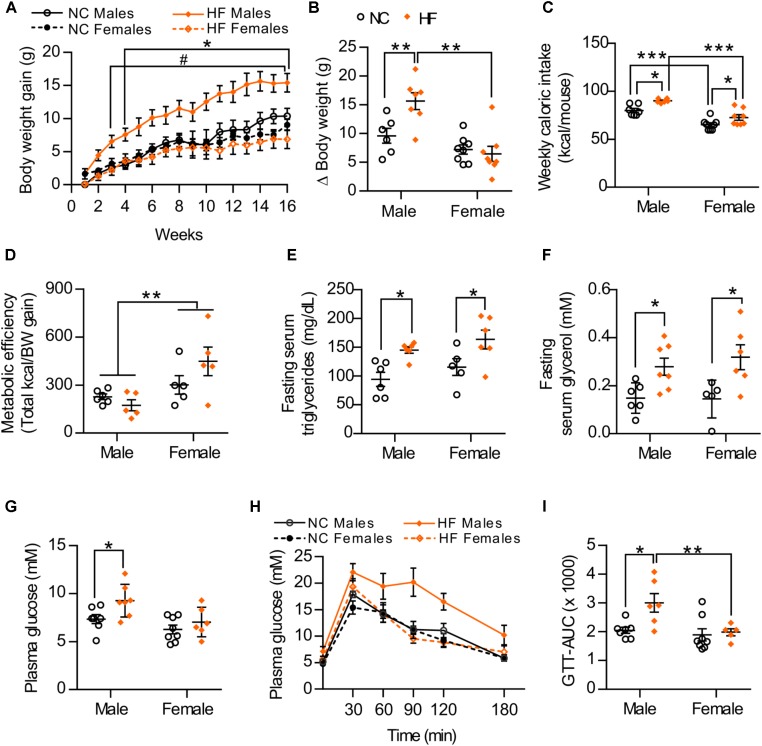
Sex differences in the development of diet-induced obesity and related metabolic disorders. **(A)** Body weight gain during 16 weeks of NC or HF feeding, **(B)** Summarized weight gain over the course of 0–14 weeks. **(C)** Total kcal of food per mouse consumed from weeks 7 to 13. **(D)** Metabolic efficiency (energy intake consumed between weeks 7 and 13 divided by the body weight gain over the same period of time). **(E,F)** Fasting serum triglycerides **(E)** and glycerol **(F)** after 16 weeks of NC or HF diet. **(G)** Glucose levels of male and female mice after 4-h fasting. **(H)** Glucose tolerance of male and female mice was assessed by intraperitoneal glucose tolerance test after 16-h fasting. **(I)** Area under the curve (AUC). Data in all panels are expressed as mean ± SEM. **(A)** ^∗^*P* < 0.05 when compared to NC group (same sex); ^#^*P* < 0.05 when compared to male mice (same diet), *post hoc* Bonferroni-corrected *t*-tests when a statistical significance was detected by two-way ANOVA model. **(B–I)** ^∗^*P* < 0.05, ^∗∗^*P* < 0.01, ^∗∗∗^*P* < 0.001, *post hoc* Bonferroni-corrected *t*-tests when statistical significance was detected by the two-way ANOVA model.

To examine sex-related differences to diet-induced obesity and insulin resistance, age-matched male and female mice were monitored while being maintained on a normal chow (NC) or a high-diet (HF) diet for 16 weeks. At the end of the diet period, no significant differences in body weight gain were observed between NC-fed male and female mice. However, upon exposure to HF diet, male mice gained significantly more weight than did the females (Figures 1A,B). Accordingly, HF-fed males presented increased weights of white adipose tissue depots, liver and heart (Table 1), which may contribute to the greater body weight gain of these mice in comparison to NC-fed males or HF-fed females (Table 1). No increase in fat accumulation or in the weight of other tissues was detected in females with HF feeding (Table 1). HF diet feeding increased energy intake in both male and female mice, although females had lower energy intake and higher metabolic efficiency than their diet-matched male counterparts (Figures 1C,D). Moreover, circulating levels of serum triglycerides and glycerol were similarly increased in both HF-fed female and male mice compared to NC groups (Figures 1E,F). In turn, plasma glucose levels were elevated in HF *vs*. NC-fed male mice whereas no diet effect was observed in females (Figure 1G). HF feeding also reduced glucose tolerance in male mice but not in females (Figures 1H,I), indicating that whole-body glucose homeostasis was maintained in HF-fed females despite impaired lipid metabolism.

**Table 1:** Body and tissue weights of male and female mice after 16 weeks of NC or HF diet.

	NC male	NC female	HF male	HF female
Body weight (g)	31.3 0.8	27.3 1.1	44.4 1.2*	30.2 1.0#
pgWAT (mg)	1177.4 104.6	937.9 127.4	1839.1 197.7*	1176.9 203.8#
rWAT (mg)	561.2 43.1	552.9 84.6	881.7 86.1*	450.5 119.4#
BAT (mg)	169.1 19.9	124.3 4.1	189.2 21.2	93.7 7.7#
Liver (mg)	1177.6 85.8	982.9 67.0	1517.9 93.3*	908.2 69.0#
Heart (mg)	103.4 2.4	100.8 3.0	134.9 4.2*	109.4 4.1#
TA (mg)	39.2 1.4	34.3 1.3	42.8 2.2	37.8 2.4
EDL (mg)	9.9 0.5	8.3 0.2	10.4 0.5	8.8 0.3
Gastrocnemius (mg)	118.1 2.5	97.8 5.3	130.9 4.3	100.0 0.7
Plantaris (mg)	14.3 0.7	11.9 0.6	15.6 0.7	11.8 0.4
Soleus (mg)	7.1 0.3	7.3 0.8	8.2 0.5	6.6 0.4


*pgWAT, perigonadal white adipose tissue; rWAT, retroperitoneal adipose tissue; BAT, brown adipose tissue; TA, Tibialis anterior; EDL, extensor digitorium longus. Data are expressed as mean ± SEM. *n* = 7 NC-males, *n* = 8 NC-females, *n* = 7 HF-males, and *n* = 6 HF-females. ^∗^*P* < 0.05 when compared to NC group (same sex); ^#^*P* < 0.05 when compared to male mice (same diet), *post hoc* Bonferroni-corrected *t*-tests when a statistical significance was detected by two-way ANOVA model.*

### HF-Fed Females Exhibit Greater Insulin Sensitivity Than Male Mice

**FIGURE 2:**
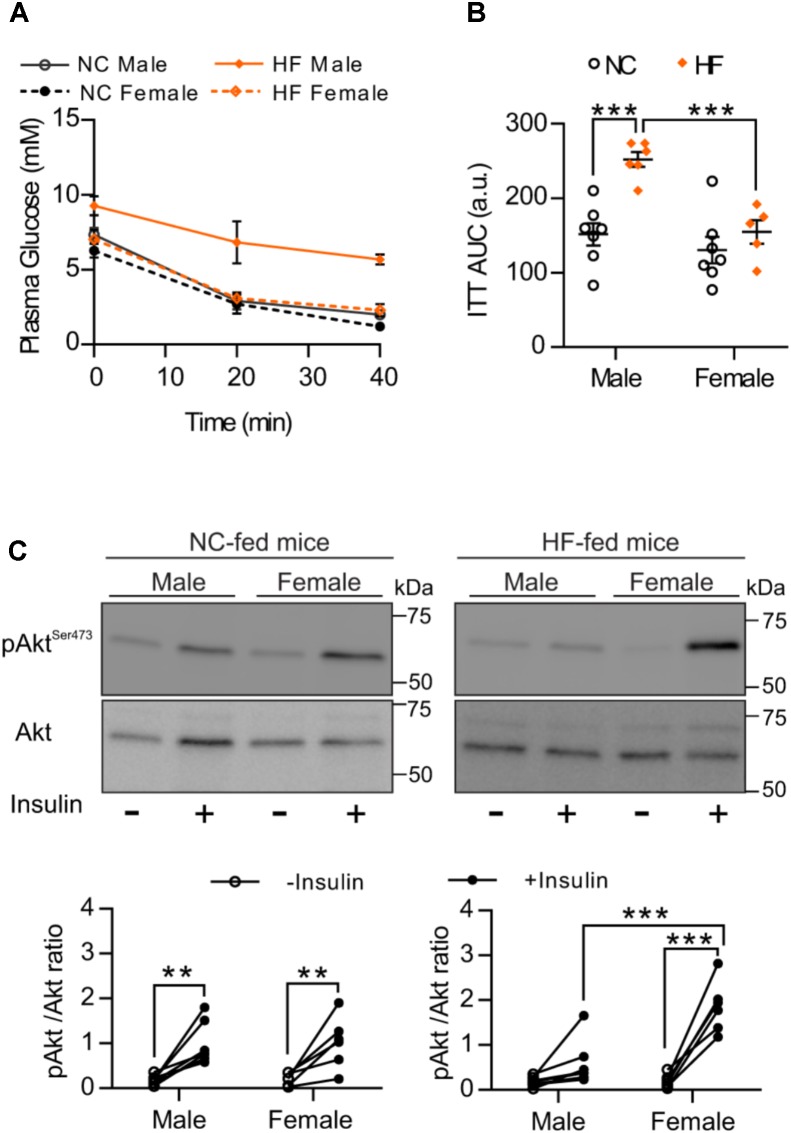
HF-fed female mice showed increased insulin sensitivity compared to males. **(A)** Insulin sensitivity of male and female mice was examined by intraperitoneal insulin test after 4-h fasting. **(B)** Area under the curve (AUC). **(C)** pSer473-Akt was assessed in EDL muscle of NC and HF-fed male and female mice before and after *in vivo* insulin injection. Representative Western blots images (top panels) and quantitative analysis (bottom panels) of pSer473-Akt and total Akt levels. Results are expressed relative to total Akt levels. *n* = 7 NC-male mice, *n* = 7 HF-male mice, *n* = 6 NC-female mice, *n* = 6 HF-female mice. Data in all panels are expressed as mean ± SEM. ^∗∗^*P* < 0.01, ^∗∗∗^*P* < 0.001; *post hoc* Bonferroni-corrected *t*-tests when statistical significance was detected by the two-way ANOVA model.

To analyze whether greater glucose tolerance and lower glucose levels of HF-fed females result from differences in insulin sensitivity, we conducted an ITT. Similar to the GTT results, we observed that HF feeding leads to impaired whole-body insulin responsiveness in male but not in female mice. No difference was detected between NC-fed male and females (Figures 2A,B). Given that skeletal muscle is one of the major regulators of systemic insulin sensitivity through insulin-mediated glucose uptake, we next examined *in vivo* muscle insulin sensitivity. Consistent with the responses to the ITT, phosphorylation of insulin downstream target, Akt, increased equivalently in skeletal muscle from NC-fed males and females after an i.p. insulin injection (Figure 2C). In contrast, insulin-stimulated Akt phosphorylation was significantly greater in skeletal muscle of HF-fed females than HF-fed males (Figure 2C), confirming that females retained muscle insulin sensitivity under high-fat feeding conditions.

### Higher Insulin Sensitivity in HF-Female Mice Is Not Attributable to Increased Muscle Capillarization

**FIGURE 3:**
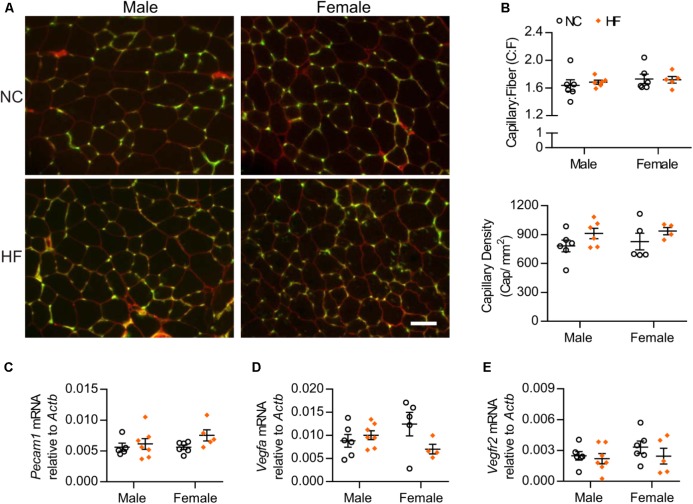
Skeletal muscle capillarization was unaltered. **(A)** Images of EDL muscle stained with *G. simplicifolia* lectin-FITC (green) and Rhodamine Wheat Germ Agglutinin (red) to visualize capillaries and outline muscle fibers, respectively (scale bar = 50 μm). **(B)** Capillary-to-muscle fiber (C:F) ratios and capillary density were calculated from 3 and 6 independent fields of view per mouse. **(C–E)** EDL gene expression analysis by qPCR. Data in all panels are expressed as mean ± SEM.

Considering that capillary density correlates with muscle insulin sensitivity, we assessed capillary number of male and female mice. We observed that neither the capillary-to-muscle fiber (C:F) ratio nor the capillary density differed between sexes or was influenced by diet (Figures 3A,B). In agreement with the morphological data, gene expression analysis of skeletal muscle indicated no significant diet or sex differences in mRNA levels of the endothelial cell marker *Pecam1* (Figure 3C). We also found no diet- or sex-related differences in mRNA levels of the pro-angiogenic factor, *Vegfa* or one of its receptors (VEGF receptor 2) (Figures 3D,E). Altogether, these data suggest that the greater *in vivo* muscle insulin sensitivity of HF-fed female mice was not due to differences in capillary number.

### Female Mice Showed Preserved Adipose Tissue Functions After 16 Weeks of HF Diet

**FIGURE 4:**
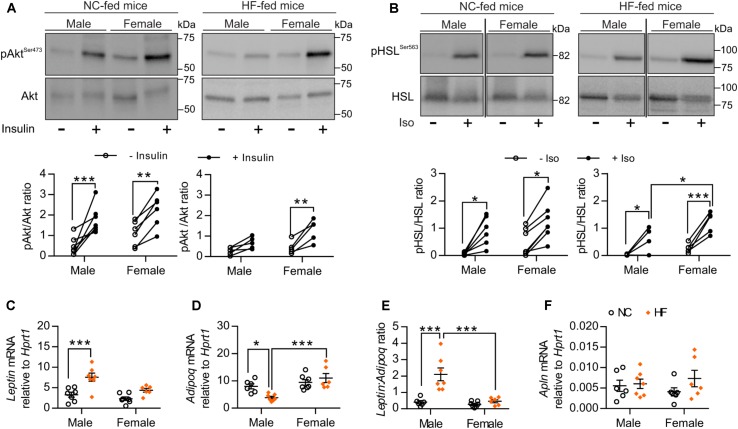
HF-fed female mice exhibited improved adipose tissue function. **(A)** Representative Western blot images (top) and quantitative analysis (bottom) of pSer473-Akt and total Akt levels in pgWAT after *ex vivo* incubation in the absence or presence of 25 mU/mL insulin for 30 min. Results are expressed relative to total Akt levels. *n* = 7 NC-male mice, *n* = 5 HF-male mice, *n* = 6 NC-female mice, *n* = 5 HF-female mice. **(B)** Representative Western blot images (top) and quantitative analysis (bottom) of pSer563-HSL and total HSL levels in pgWAT after *ex vivo* incubation in the absence or presence of 10 μmol/L isoproterenol for 30 min. Results are expressed relative to total HSL levels. *n* = 6 NC-male mice, *n* = 4 HF-male mice, *n* = 7 NC-female mice, *n* = 5 HF-female mice. **(C–F)** pgWAT gene expression analysis by qPCR. Data in all panels are expressed as mean ± SEM. ^∗^*P* < 0.05, ^∗∗^*P* < 0.01, ^∗∗∗^*P* < 0.001, *post hoc* Bonferroni-corrected *t*-tests when statistical significance was detected by the two-way ANOVA model.

We next examined sex-related differences in adipose tissue function, based on the knowledge that adipose tissue plays a major role in regulating whole-body homeostasis and that dysfunction of adipose tissue induced by obesity facilitates the development of whole-body metabolic disturbances (Rosen and Spiegelman, 2006). First, we evaluated *ex vivo* insulin sensitivity of pgWAT from NC and HF-fed male and female mice. No difference in adipose insulin sensitivity was detected between male and female mice fed a NC diet (Figure 4A). Similar to what we observed in the skeletal muscle, Akt phosphorylation in response to insulin was retained in pgWAT from HF-fed female mice whereas this response was impaired in HF-fed males (Figure 4A). *Ex vivo* stimulation of pgWAT with the pro-lipolytic beta-adrenergic agonist isoproterenol was performed as an additional test of adipose function. Analysis of isoproterenol-stimulated phosphorylation of the hormone-sensitive lipase (HSL) indicated that pgWAT from NC-fed males and females had similar sensitivity to isoproterenol. However, HF-fed females showed enhanced sensitivity compared to HF-fed male counterparts (Figure 4B). Given that adipose dysfunction is associated with an imbalance in the expression of adipokines and peptides (Romacho et al., 2014), which can modulate whole-body glucose and insulin tolerance, we evaluated the expression of *Leptin*, *Adiponectin*, and *Apelin* in the pgWAT. While HF feeding resulted in imbalanced adipokine levels in male mice, with increased *Leptin* and decreased *Adiponectin* mRNA levels, it failed to elicit any changes in the levels of these adipokines in adipose tissue of females (Figures 4C,D). Additionally, HF-females showed increased levels of *Adiponectin* compared to males (Figure 4D). Accordingly, HF-fed males presented higher *leptin:adiponectin* mRNA ratio compared to NC-fed males and HF-fed females, confirming that high-fat feeding provokes an imbalance in adipokine expression only in male adipose tissue (Figure 4E). No significant diet- or sex-differences were detected in pgWAT mRNA levels of *Apelin* (a peptide that can exert metabolic influences) (Figure 4F).

**FIGURE 5:**
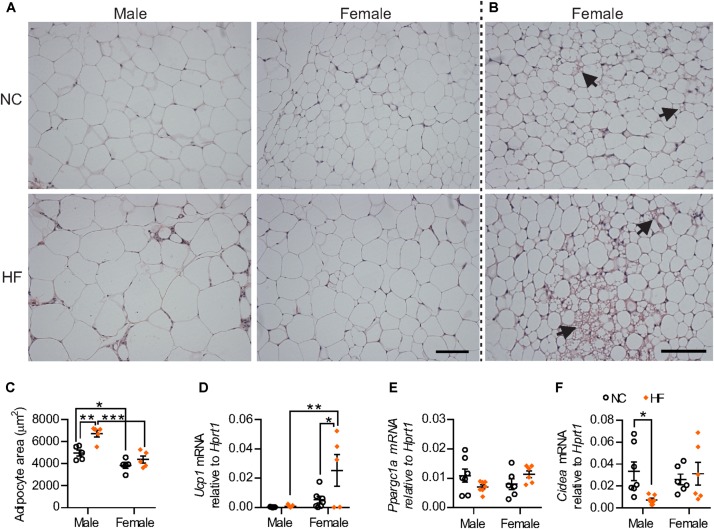
Distinct phenotype of visceral adipose tissue from female mice. **(A)** Representative images of hematoxylin and eosin-stained pgWAT from NC and HF-fed male and female mice. Scale bar = 100 μm. **(B)** Examples of adipose browning (right panels) observed in some regions of adipose within both NC and HF fed females. **(C)** Mean adipocyte area. **(D–F)** mRNA for browning markers *Ucp1* **(D)**, *Ppargc1a* **(E)** and for *Cidea* **(F)** relative to *Hprt1* in pgWAT. Data are expressed as mean ± SEM; ^∗^*P* < 0.05, ^∗∗^*P* < 0.01, ^∗∗∗^*P* < 0.001, *post hoc* Bonferroni-corrected *t*-tests when statistical significance was detected by the two-way ANOVA model.

HF diet increased adipocyte size only in male mice whereas adipocytes from female pgWAT were smaller when compared to male mice regardless of diet (Figures 5A–C). Remarkably, cells with multilocular fat droplets were observed in pgWAT from some NC and HF-fed females but not in any of the males (Figure 5B). Gene expression analysis revealed increased mRNA levels of the thermogenic gene *Ucp-1* in HF-females. There were no sex-differences in the expression of *Ppargc1a* and *Cidea*, genes encoding other regulators of thermogenesis, although reduced levels of *Cidea* expression were detected in males following HF diet (Figures 5D–F). Taken together, these findings demonstrate that, while HF diet feeding leads to adipose tissue dysfunction in male mice, this effect is not observed in females.

### HF-Fed Females Show Higher Adipose Tissue Vascularity

**FIGURE 6:**
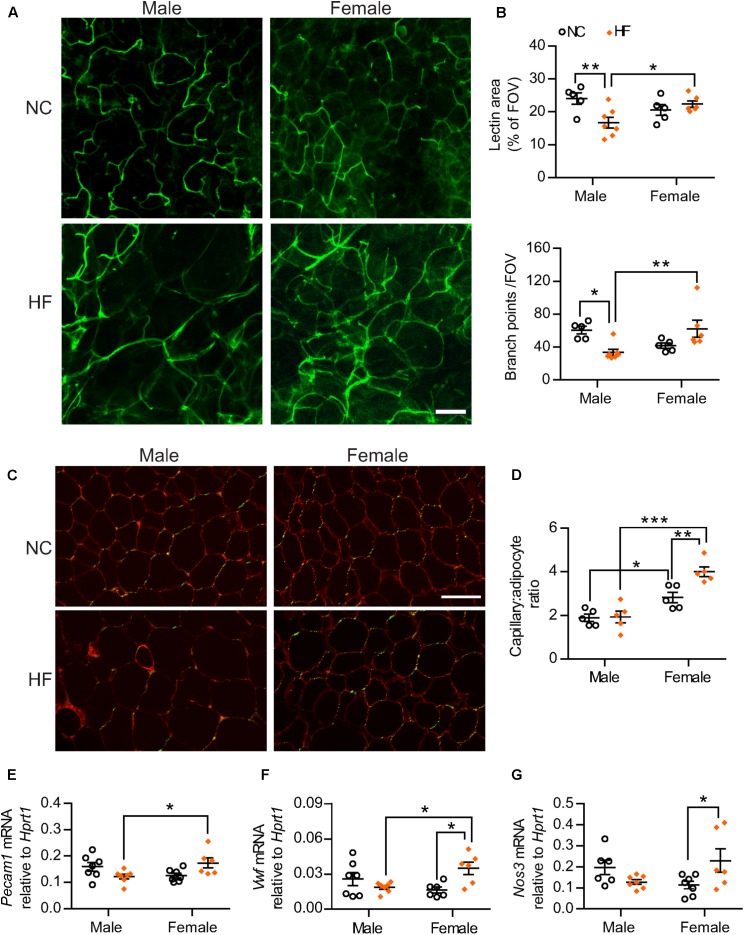
Adipose tissue angiogenesis occurred in HF-fed females. **(A)** Representative confocal images of pgWAT whole-mount staining with *Griffonia simplicifolia* lectin (green) (scale bar = 100 μm). **(B)** Lectin area and capillary branch density were quantified from confocal images (FOV = field of view). **(C,D)** *Griffonia simplicifolia* lectin (green) and Wheat germ agluttinin (red) staining of paraffin-sectioned adipose tissue **(C)** was utilized to calculate capillary to adipocyte ratio **(D)**. Scale bar = 100 μm. **(E–G)** pgWAT gene expression analysis by qPCR. Data in all panels are expressed as mean ± SEM. ^∗^*P* < 0.05, ^∗∗^*P* < 0.01, ^∗∗∗^*P* < 0.001, *post hoc* Bonferroni-corrected *t*-tests when statistical significance was detected by the two-way ANOVA model.

Given the known importance of adequate vascularization in preventing diet-induced adipose tissue dysfunction and linkage with browning of white adipose tissue (Sun et al., 2012; Crewe et al., 2017), we analyzed the adipose tissue microvasculature of male and female mice. Confocal fluorescent imaging of pgWAT revealed that HF-fed males displayed a marked reduction in vascular density when compared to NC-males (Figures 6A,B). In contrast, no diet effect was observed in the adipose tissue from females. The adipose tissue from HF-fed females thereby showed greater vascular content when compared to male counterparts, as demonstrated by higher vascular area and number of vessel branch points (Figures 6A,B). To better control for the influence of adipocyte size on the assessment of vascularization, capillary number relative to adipocyte number also was quantified in pgWAT tissue sections (Figures 6C,D). Capillary number per adipocyte was higher in pgWAT of females regardless of the diet. Additionally, HF increased the capillary number per adipocyte in females whereas no diet effect was detected in pgWAT from male mice. In line with the increased capillary number detected in HF-females, gene expression analysis indicated higher mRNA levels of endothelial cell markers *Pecam1* and von Willebrand Factor (*Vwf*) in pgWAT of these mice than their diet-matched male counterparts (Figures 6E,F). In addition, a diet effect was observed for *Vwf* and endothelial nitric oxide synthase (*Nos3*) in females, with elevated mRNA levels of both genes detected in HF-females (Figures 6F,G), corroborating a greater endothelial cell content in the adipose tissue of these mice.

Next, we considered whether the greater endothelial cell content within pgWAT of HF-fed females was a reflection of the lower weight gain observed in those mice compared to their male counterparts. Therefore, we assessed the pgWAT vascularization in a second cohort of mice, in which weight-matched male and female mice had an acute 7-day exposure to HF feeding, as short diet periods can also initiate adipose expansion leading to impaired glucose tolerance in male mice (Williams et al., 2014). It is notable that these females were older and presented greater adiposity, particularly of visceral depots, compared to the weight-matched male mice (Supplementary Table 1). Regardless, confocal and histological analysis of capillary to adipocyte ratio confirmed the higher adipose tissue vascularity within pgWAT of females (Supplementary Figures 1A–D). Moreover, gene expression analysis of pgWAT detected increased *Vwf* mRNA levels. *Pecam1* expression also tended to be higher in females (*P* = 0.07), although not reaching statistical significance with this sample size (Supplementary Figures 1E,F). Thus, our findings in both cohorts of mice indicate that pgWAT expansion of females is accompanied by a concomitant growth of its vascular network contrasting with the compromised pattern of vascularization observed in male pgWAT upon HF feeding.

### Pro-angiogenic Environment Was Detected Within Adipose Tissue From HF-Fed Female Mice

**FIGURE 7:**
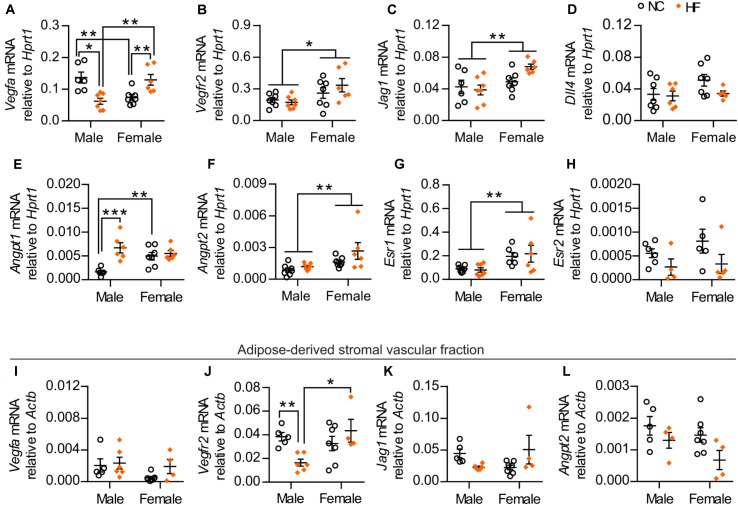
HF-fed female mice show higher levels of pro-angiogenic markers and estrogen receptor 1 in pgWAT. **(A–H)** pgWAT gene expression analysis by qPCR. **(I–L)** qPCR analysis of the adipose tissue-derived stromal vascular fraction. Data in all panels are expressed as mean ± SEM. ^∗^*P* < 0.05, ^∗∗^*P* < 0.01 for main sex effect by two-way ANOVA model **(B,C,F,G)**, ^∗^*P* < 0.05, ^∗∗^*P* < 0.01, ^∗∗∗^*P* < 0.001, *post hoc* Bonferroni-corrected *t*-tests when a statistical sex x diet interaction significance was detected by the two-way ANOVA model **(A,E,J)**.

To investigate potential mechanisms that could account for the higher adipose vascular density of HF-fed female mice, we assessed the gene expression of key pro-angiogenic factors in pgWAT. While HF diet feeding reduced *Vegfa* mRNA in adipose tissue from males, it increased its expression in females. In addition, NC-fed females demonstrated lower *Vegfa* mRNA compared to their male counterparts, while this pattern was reversed by HF diet (Figure 7A). A main effect of sex was also detected in the gene expression of additional pro-angiogenic factors, including VEGF receptor-2 (*Kdr*), the Notch ligand Jagged-1 (*Jag1*) and angiopoietin-2 (*Angpt2*) (Figures 7B,C,F). Conversely, no sex or diet effect was observed in the mRNA levels of *Dll4* (encoding Delta-like 4), a Notch ligand that acts as a negative regulator of angiogenic sprouting (Figure 7D) (Lobov et al., 2007). In contrast, HF diet increased the expression of angiopoietin-1 (*Angpt1* – a factor that promotes capillary stabilization) only in male mice, although NC-fed females showed enhanced levels when compared to male counterparts (Figure 7E). Given that *Vegfa* levels in adipose tissue can be regulated by estrogen receptor signaling (Fatima et al., 2017), we assessed the mRNA levels of estrogen receptor 1 (*Esr1*) and estrogen receptor 2 (*Esr2*). Under our experimental conditions, a main effect of sex was detected in *Esr1* expression while no statistically significant effect of sex or diet was detected in *Esr2* expression (Figures 7G,H).

To determine if stromal vascular cells contribute to the observed changes in the gene expression of the pro-angiogenic factors *Vegfa*, *Vegfr2*, *Jag1* and *Angpt2* within the pgWAT, we assessed the mRNA levels of these genes in the adipose-derived SVF of the same mice. No statistically significant sex or diet effects were observed in the expression of *Vegfa* or *Jag1* (Figures 7I,K). In contrast, expression of *Vegfr2* was significantly higher in HF-fed females compared to male counterparts (Figure 7J). Further, a main diet effect on the mRNA levels of *Angpt2* was detected (Figure 7L). Taken together, these findings indicate that adipose tissue from females had enriched levels of factors with pro-angiogenic activity, arising from both adipose-derived SVF and adipocytes, which can contribute to the greater adipose vascularity in these mice.

## Discussion

**FIGURE 8:**
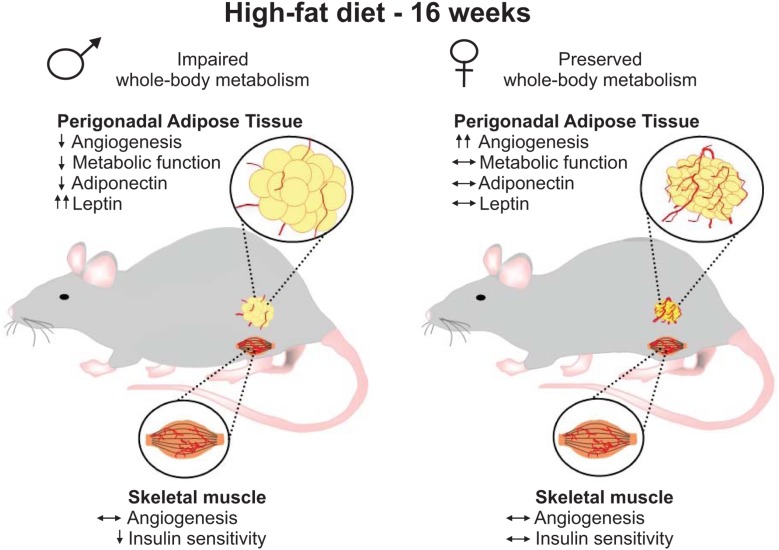
Schematic depiction of results. After 16 weeks of high-fat diet, females gained less weight and showed preserved whole-body metabolism when compared to male mice. At the tissue level, male and female mice demonstrated similar skeletal muscle capillarization. While HF-fed males presented muscle insulin resistance, no impairment in muscle insulin sensitivity was detected in female mice. Higher vascular density was detected in perigonadal adipose tissue from HF-fed females, which was associated with greater mRNA levels of pro-angiogenic mediators and estrogen receptor 1 and preserved tissue metabolic functions and distinct adipokines expression. Conversely, HF-fed males showed decreased adipose vascularity and adipose tissue dysfunction.

In this study, we provide the first evidence of sex-differences in the vascular remodeling of the perigonadal adipose tissue of mice under HF feeding conditions. Resistance to the development of disturbed glucose homeostasis upon HF-feeding, as was recapitulated in our current study, is a hallmark feature that distinguishes females from males not only in rodent obesity models (Macotela et al., 2009; Medrikova et al., 2012; Pettersson et al., 2012) but also is observed in pre-menopausal females in human studies (Frias et al., 2001; Varlamov et al., 2014). Given that glucose homeostasis largely reflects functions of key insulin target tissues, skeletal muscle and adipose, and that insulin delivery is significantly influenced by capillary network density, the primary purpose of this study was to identify potential sex differences in the influence of prolonged high-fat diet on vascularization in these tissues. While no differences in capillarization were observed in skeletal muscle, higher vascular density was a striking phenotypic characteristic in female perigonadal adipose tissue that contrasted with the distinct lack of angiogenesis observed in male adipose tissue. Interestingly, the greater endothelial cell content of pgWAT of females was also observed in an independent cohort of weight-matched male and female mice, reinforcing the concept of sex-dependent differences of adipose vascular content. We also found that vascular remodeling in this adipose tissue depot of HF-fed females coincided with improved maintenance of metabolic functions of this tissue and whole-body homeostasis under HF feeding conditions (Figure 8). These results lead us to postulate that the greatest vessel density within the perigonadal adipose tissue may contribute to the lower susceptibility of female mice in developing obesity-driven metabolic disturbances.

### Greater Vascular Growth of Female Adipose Tissue Under HF Conditions May Be Explained by Higher Expression of Pro-angiogenic Factors

Our findings provide compelling evidence that the greater vascular density of perigonadal adipose tissue from HF-fed female mice is matched with enhanced expression of factors with pro-angiogenic activity. In particular, we show that HF diet exerted opposing sex-dependent influences on adipose tissue *Vegfa* mRNA level, which declined in males but increased in females, such that perigonadal adipose tissue from HF-fed females displayed increased expression of *Vegfa* compared to male counterparts. These findings suggest that angiogenesis-associated differences between male and female mice may result from HF-induced changes in VEGFA expression. Consistently, prior reports indicated that VEGFA level is indeed a critical determinant of adipose vascularization in male mice (Elias et al., 2012; Sun et al., 2012, Sung et al., 2013). Furthermore, our data indicate that the elevated level of *Vegfa* mRNA detected in the whole adipose tissue of HF-fed females likely originates from adipocytes, since this pattern of expression was not detected in the SVF. Recent data show that 17 β-estradiol can regulate VEGFA expression in adipocytes and adipose tissue depots through its interaction with estrogen receptor 1 (ESR1), with female ESR1 knockout mice thereby presenting lower levels of VEGFA and VEGFR2 in visceral adipose tissue (Fatima et al., 2017). Importantly, a sex-related difference was also detected in the expression of the main VEGF receptor, VEGFR2, with females having higher mRNA levels of the gene encoding this receptor. Thus, it is likely that sexually dimorphic differences in VEGFA signaling account for the higher adipose tissue angiogenesis detected in female mice under HF feeding conditions.

Although VEGFA is by far the most investigated pro-angiogenic regulator of adipose tissue angiogenesis, regulation of vascular remodeling is also influenced by different signaling pathways, such as those mediated by Notch and angiopoietin ligands (Maisonpierre et al., 1997; Blanco and Gerhardt, 2013). Of note, higher mRNA levels of *Jagged-1* (Notch ligand) and *Angiopoietin-2* were detected in the perigonadal adipose tissue from HF-fed females. Previous studies have shown that Jagged-1 and angiopoietin-2 exert pro-angiogenic influences in adipose tissue and the expression of both genes can be regulated by estradiol (Ye et al., 2002; Soares et al., 2004; Urs et al., 2012; An et al., 2017). Therefore, multiple pro-angiogenic pathways are represented to a greater extent within the perigonadal adipose tissue of HF-fed females than in their male counterparts and are likely contributors to the higher vascular density observed in the HF-fed female mice. In the future, it will be interesting to unravel the underlying mechanisms of increased expression of angiogenic mediators and decipher the angiogenic signals that are crucial to provoke improved angiogenic responses in females.

### Increased Vascular Density in Adipose Tissue of HF-Fed Female Mice Was Associated With Preservation of Adipose Tissue Functions and Peripheral and Systemic Insulin Sensitivity

Concomitant with the angiogenic phenotype, HF-females exhibited smaller adipocytes, improvements in their insulin sensitivity and adipokine mRNA profiles and augmented browning of the perigonadal adipose tissue, in comparison to their male counterparts. These findings are in line with sex differences described previously. Indeed, studies have indicated that female adipocytes have higher insulin sensitivity and enhanced thermogenic reprogramming compared with male adipocytes (Rodriguez-Cuenca et al., 2002; Macotela et al., 2009; Grove et al., 2010; Nookaew et al., 2013; Kim et al., 2016). It is conceivable that the “healthier” phenotype of the white adipose tissue observed in females upon HF feeding is actively promoted by the differences in angiogenesis within this tissue in females *vs*. males. Numerous preclinical and clinical studies have documented that appropriate adipose angiogenesis is of vital importance for the maintenance of adipose tissue functions during obesity (Pasarica et al., 2009; Elias et al., 2012; Sun et al., 2012; Sung et al., 2013; An et al., 2017). Interestingly, increased adipose angiogenesis has been linked to enhanced adiponectin expression (Sung et al., 2013) and plays a crucial role in determining browning (Sun et al., 2012; Robciuc et al., 2016; Seki et al., 2018). It has been also shown that the microvascular expansion provoked by VEGF overexpression precedes the appearance of beige (UCP1-positive cells) adipocytes (Park et al., 2017). In addition to the studies linking the activation of thermogenic and browning programs in white adipose tissue depots of male mice with VEGF-A/VEGFR2 pathway (Sun et al., 2011; Robciuc et al., 2016; Park et al., 2017; Seki et al., 2018), other pro-angiogenic factors upregulated in female adipose tissue in our study are known mediators of the fate of pre-adipocytes. In particular, Jagged-1 promotes proliferation of pre-adipocytes through the inactivation of Notch signaling (Urs et al., 2012), which has been associated with increased *Ucp1* expression and browning of adipocytes (Bi et al., 2014). Thus, given the published evidence that remodeling of adipose tissue vasculature and upregulation of pro-angiogenic factors are sufficient to modulate pre-adipocyte fate and adipose tissue phenotype, we postulate that vascular growth in the perigonadal adipose tissue of females sustains adipocyte health under HF feeding conditions.

Our results, as well as previous studies (Macotela et al., 2009; Grove et al., 2010; Pettersson et al., 2012), also demonstrate that females were resistant to metabolic disturbances associated with a HF diet. In this context, it is notable that the higher insulin sensitivity observed in HF-female skeletal muscles occurred independently of a change in muscle capillarity. Considering that adipose tissue plays a crucial role in the control of whole-body metabolism, not only through managing lipid storage but also via the release of adipokines that exert functional influences on peripheral tissues, it is feasible that the higher insulin sensitivity is a direct reflection of the improved crosstalk between adipose and muscle tissues. This is consistent with the absence of impairment in adiponectin or leptin expression, both of which regulate muscle insulin sensitivity (Li et al., 2017) although we cannot exclude inherent differences in skeletal myocyte insulin responsiveness. Additionally, our findings could be confounded by the fact that females gain less weight than male mice under HF feeding conditions. However, it is noteworthy that the studies describing the impact of increased angiogenesis within visceral adipose tissue of HF-fed male mice report not only local changes in tissue functions but also a reduction in the development of systemic and peripheral metabolic disturbances (Sun et al., 2012; Sung et al., 2013; Robciuc et al., 2016), reinforcing the idea that adipose angiogenesis ultimately influences metabolic homeostasis. Indeed, the increased adipose browning as a consequence of increased vascular density of HF-fed females may result in higher metabolic turnover of fat in these mice. Additionally we and others (Pujol et al., 2003) have observed higher lipolytic sensitivity in female adipocytes, which in conjunction with the browning phenotype may provide resistance to the development of diet-induced metabolic abnormalities through enhancing metabolic efficiency and decreasing fat accumulation. Thus, future studies will be required to address whether expansion of the adipose vasculature constitutes either a contributing or a primary mechanism through which systemic energy homeostasis is preserved in response to high-fat diet.

## Conclusion

In summary, our findings highlight that females on a high-fat diet have greater vascularity in perigonadal adipose tissue than male mice. Moreover, we present several lines of evidence to support the perspective that increased adipose vasculature in females is associated with lower fat accumulation, increased adipose tissue browning, preserved adipose tissue functions, whole-body glucose metabolism and greater muscle insulin sensitivity. However, no sex-difference was detected in plasma lipids or in muscle capillarization. Thus, the distinct angiogenic response elicited by high-fat diet in this adipose tissue depot of females could help to explain why female mice are more resistant to diet-induced obesity and less prone to develop its related metabolic abnormalities. These findings highlight the importance of considering sex-differences in adipose angiogenesis in future studies assessing obesity-associated disorders of male and females.

## Author Contributions

MR, GA, ER, and TH conceived the study. MR, GA, ER, and TH designed the experiments. MR, GA, OR, EN, ER, and TH performed the experiments. MR, GA, OR, and TH analyzed the data. MR and TH wrote the manuscript with contributions from all authors.

## Conflict of Interest Statement

The authors declare that the research was conducted in the absence of any commercial or financial relationships that could be construed as a potential conflict of interest.
