# Supplementary material for: Female Mice Have Higher Angiogenesis in Perigonadal Adipose Tissue Than Males in Response to High-Fat Diet
Source: Front Physiol. 2018 Oct 23;9:1452. doi: 10.3389/fphys.2018.01452 (PMC6206240; doi:10.3389/fphys.2018.01452)
Supplement: Supplementary file 1 [file Table_1.DOC]

**Supplemental Table 1. Characteristics of 2^nd^ cohort of mice**

|  | **Male** | **Female** |
| --- | --- | --- |
| Age (weeks) | 17.3±2.2 | 24.8±1.3 |
| Body weight (g) | 31.5±1.3 | 33.43±1.5 |
| pgWAT (mg) | 1160±167.6 | 1815±179.4* |
| rWAT (mg) | 360±33.9 | 670±119.2* |
| BAT (mg) | 120±31.4 | 150±5.8 |
| Subcutaneous (mg) | 1158±310.7 | 1483±152.2 |

Abbreviations: pgWAT, perigonadal white adipose tissue; rWAT, retroperitoneal adipose tissue; BAT, brown adipose tissue. Data are expressed as mean ± SEM. *n*=4 **P*<0.05 calculated with two-tailed unpaired *t* test.
