# Supplementary material for: Female Mice Have Higher Angiogenesis in Perigonadal Adipose Tissue Than Males in Response to High-Fat Diet
Source: Front Physiol. 2018 Oct 23;9:1452. doi: 10.3389/fphys.2018.01452 (PMC6206240; doi:10.3389/fphys.2018.01452)
Supplement: Supplementary file 2 [file Data_Sheet_1.PDF]

Suplemental Figure 1

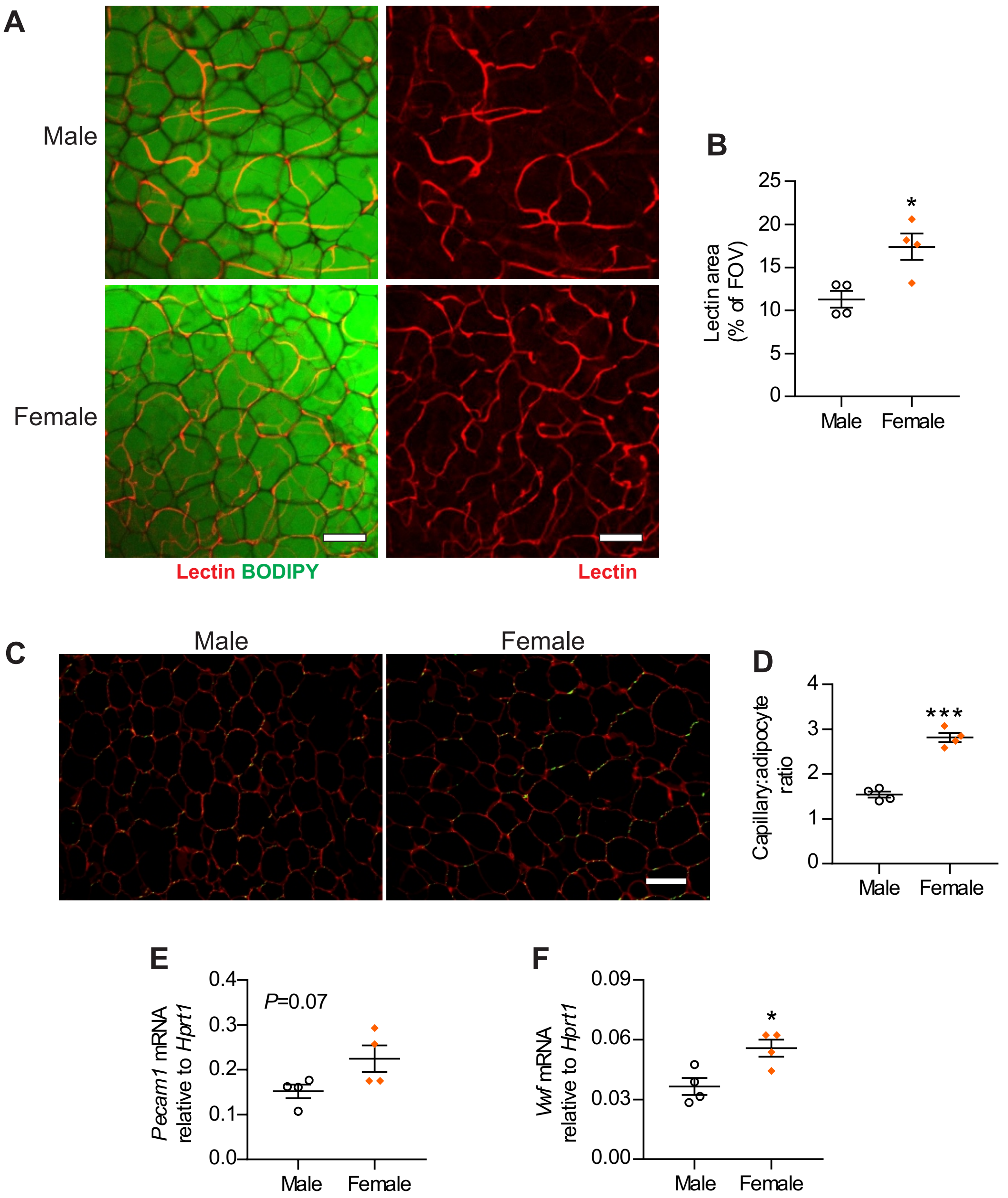

Supplemental Figure 1. Weight-matched females exhibit greater perigonadal adipose vascularity. (A) Representative confocal images of pgWAT whole-mount staining with BODIPY 493/503 (green) and *Griffonia Simplicifolia* lectin (red; x10 magnification; scale bar = 100 µm). **(B)** Lectin area quantified from confocal images (FOV = field of view). **(C-D)** *Griffonia Simplicifolia* lectin (green) and Wheat germ agluttinin (red) staining of paraffin-sectioned adipose tissue (C) was utilized to calculate capillary to adipocyte ratio (D). Scale bar = 100 µm. **(E-F)** pgWAT gene expression analysis by qPCR. Data in all panels are expressed as mean ± SEM. *P<0.05, ***P<0.001, calculated with two-tailed unpaired *t* test.
